# Extragradient subgradient methods for solving bilevel equilibrium problems

**DOI:** 10.1186/s13660-018-1898-1

**Published:** 2018-11-26

**Authors:** Tadchai Yuying, Bui Van Dinh, Do Sang Kim, Somyot Plubtieng

**Affiliations:** 1 Department of Mathematics, Faculty of Science, Naresuan University, Phitsanulok, Thailand; 2 Faculty of Information Technology, Le Quy Don Technical University, Hanoi, Vietnam; 3 Department of Applied Mathematics, Pukyong National University, Busan, Korea; 4 Center of Excellence in Nonlinear Analysis and Optimization, Faculty of Science, Naresuan University, Phitsanulok, Thailand

**Keywords:** 47J25, 65K10, 65K15, 90C25, Bilevel equilibrium problems, Extragradient Subgradient-Halpern methods, Armijo line search, Strong convergence

## Abstract

In this paper, we propose two algorithms for finding the solution of a bilevel equilibrium problem in a real Hilbert space. Under some sufficient assumptions on the bifunctions involving pseudomonotone and Lipschitz-type conditions, we obtain the strong convergence of the iterative sequence generated by the first algorithm. Furthermore, the strong convergence of the sequence generated by the second algorithm is obtained without a Lipschitz-type condition.

## Introduction

Let *C* be a nonempty closed convex subset of a real Hilbert space *H*, and let *f* and *g* be bifunctions from $H\times H$ to $\mathbb{R}$ such that $f(x,x)=0$ and $g(x,x)=0$ for all $x\in H$. The equilibrium problem associated with *g* and *C* is denoted by $EP(C,g)$: Find $x^{*}\in C$ such that

1 $$ g\bigl(x^{*}, y\bigr)\leq0\quad\text{for every } y\in C. $$

The solution set of problem (1) is denoted by *Ω*. The equilibrium problem is very important because many problems arise in applied areas such as the fixed point problem, the (generalized) Nash equilibrium problem in game theory, the saddle point problem, the variational inequality problem, the optimization problem and others.

The basic method for solving the monotone equilibrium problem is the proximal method (see [14, 16, 19]). In 2008, Tran et al. [25] proposed the extragradient algorithm for solving the equilibrium problem by using the strongly convex minimization problem to solve at each iteration. Furthermore, Hieu [9] introduced subgradient extragradient methods for pseudomonotone equilibrium problem and the other methods (see the details in [1, 8, 10, 15, 17, 22, 28]).

In this paper, we consider the equilibrium problem whose constraints are the solution sets of equilibrium problems (bilevel equilibrium problems) : Find $x^{*}\in\varOmega$ such that

2 $$ f\bigl(x^{*},y\bigr)\geq0\quad\text{for every } y\in \varOmega. $$

The solution set of problem (2) is denoted by $\varOmega^{*}$. The bilevel equilibrium problems were introduced by Chadli et al. [4] in 2000. This kind of problems is very important and interesting because it is a generalization class of problems such as optimization problems over equilibrium constraints, variational inequality over equilibrium constraints, hierarchical minimization problems, and complementarity problems. Furthermore, the particular case of the bilevel equilibrium can be applied to a real word model such as the variational inequality over the fixed point set of a firmly nonexpansive mapping applied to the power control problem of CDMA networks which were introduced by Iiduka [11]. For more on the relation of bilevel equilibrium with particular cases, see [7, 12, 21].

Methods for solving bilevel equilibrium problems have been studied extensively by many authors. In 2010, Moudafi [20] introduced a simple proximal method and proved the weak convergence to a solution of problem (2). In 2014, Quy [23] introduced the algorithm by combining the proximal method with the Halpern method for solving bilevel monotone equilibrium and fixed point problem. For more details and most recent works on the methods for solving bilevel equilibrium problems, we refer the reader to [2, 5, 24]. The authors considered the method for monotone and pseudoparamonotone equilibrium problem. If a bifunction is more generally monotone, we cannot use the above methods for solving bilevel equilibrium problem, for example, the pseudomonotone property.

Inspired by the above work, in this paper, we propose a method for finding the solution for bilevel equilibrium problems where *f* is strongly monotone and *g* is pseudomonotone and Lipschitz-type continuous. Firstly, we obtain the convergent sequence by combining an extragradient subgradient method with the Halpern method. Second, we obtain the convergent sequence without Lipschitz-type continuity on bifunction *g* by combining an Armijo line search with the extragradient subgradient method.

## Preliminaries

Let *C* be a nonempty closed convex subset of a real Hilbert space *H*. Denote that $x_{n}\rightharpoonup x$ and $x_{n}\rightarrow x$ are the weak convergence and the strong convergence of a sequence $\{x_{n}\}$ to *x*, respectively. For every $x\in H$, there exists a unique element $P_{C}x$ defined by

$$P_{C}x = \inf\bigl\{ \Vert x-y \Vert : y \in C\bigr\} . $$

It is also known that $P_{C}$ is a nonexpansive mapping from *H* onto *C*, i.e., $\|P_{C}(x)-P_{C}(y)\|\leq\|x-y\|$ $\forall x,y\in H$. For every $x\in H$ and $y\in C$, we have

$$\Vert x-y \Vert ^{2}\geq \Vert x-P_{C}x \Vert ^{2}+ \Vert y-P_{C}x \Vert ^{2}\quad \text{and}\quad \langle x-P_{C}x,P_{C}x-y\rangle\geq0. $$

A bifunction $\psi:H\times H \to\mathbb{R}$ is called:

(i) *β*-strongly monotone on *C* if
  $$\psi(x, y) + \psi(y, x) \leq-\beta \Vert x-y \Vert ^{2}\quad \forall x, y \in C; $$
(ii) monotone on *C* if
  $$\psi(x, y) + \psi(y, x) \leq0\quad \forall x, y\in C; $$
(iii) pseudomonotone on *C* if
  $$\psi(x,y)\geq0\Rightarrow\psi(y,x)\leq0, \quad \forall x, y\in C. $$

It is easy to check that the monotone bifunction implies the pseudomonotone bifunction. On the other hand, if the bifunction is pseudomonotone, then we cannot guarantee that the bifunction is monotone, for example, let $\phi(x,y)= \frac{2y-x}{1-x}$ for all $x,y\in\mathbb{R}$. It follows that *ϕ* is pseudomonotone on $\mathbb{R}^{+}\setminus\{0\}$ but *ϕ* is not monotone on $\mathbb {R}^{+}\setminus\{0\}$. Let $\psi(x, \cdot)$ be convex for every $x\in H$. For each fixed $x\in H$, the subdifferential of $\psi(x, .)$ at *x*, denoted by $\partial_{2}\psi (x, x)$, is defined by

3 $$\begin{aligned} \partial_{2}\psi(x, x) =& \bigl\{ w \in H : \psi(x,y)-\psi(x,x)\geq\langle w, y-x\rangle\ \forall y\in H\bigr\} \\ =& \bigl\{ w\in H : \psi(x,y)\geq\langle w, y-x\rangle\ \forall y\in H\bigr\} , \end{aligned}$$

studied in [13]. In this paper, we consider the bifunctions *f* and *g* under the following conditions.

### Condition A

1. $f(x, \cdot)$ is convex, weakly lower semicontinuous and subdifferentiable on *H* for every fixed $x \in H$.
2. $f (\cdot, y)$ is weakly upper semicontinuous on *H* for every fixed $y\in H$.
3. *f* is *β*-strongly monotone on *H*.
4. For each $x,y\in H$, there exists $L>0$ such that
  $$\Vert w-v \Vert \leq L \Vert x-y \Vert ,\quad \forall w\in \partial_{2}f(x,x),v\in \partial_{2}f(y,y). $$
5. The function $x \mapsto\partial_{2}f(x,x)$ is bounded on the bounded subsets of *H*.

### Condition B

1. $g(x, \cdot)$ is convex, weakly lower semicontinuous, and subdifferentiable on *H* for every fixed $x\in H$.
2. $g(\cdot, y)$ is weakly upper semicontinuous on *H* for every fixed $y\in H$.
3. *g* is pseudomonotone on *C* with respect to *Ω*, i.e.,
  $$g \bigl(x, x^{*}\bigr)\leq0,\quad \forall x\in C, x^{*}\in \varOmega. $$
4. *g* is Lipschitz-type continuous, i.e., there are two positive constants $L_{1},L_{2}$ such that $g(x,y)+g(y,z)\geq g(x,z)- L_{1}\|x-y\|^{2}-L_{2}\|y-z\|^{2}$, $\forall x,y,z\in H$.
5. *g* is jointly weakly continuous on $H \times H$ in the sense that, if $x, y \in H$ and $\{x_{n}\}, \{y_{n}\} \in H$ converge weakly to *x* and *y*, respectively, then $g(x_{n}, y_{n}) \rightarrow g(x, y)$ as $n\rightarrow+\infty$.

### Example 2.1

Let $f,g : \mathbb{R}\times\mathbb{R} \to\mathbb{R}$ be defined by $f(x,y)= 5y^{2}-7x^{2}+2xy$ and $g(x,y)=2y^{2}-7x^{2}+5xy$. It follows that *f* and *g* satisfy Condition A and Condition B, respectively.

### Lemma 2.2

([3, Propositions 3.1, 3.2])

*If the bifunction* *g* *satisfies Assumptions* (B1), (B2), *and* (B3), *then the solution set* *Ω* *is closed and convex*.

### Remark 2.3

Let the bifunction *f* satisfy Assumption A and the bifunction *g* satisfy B. If $\varOmega\neq\emptyset$, then the bilevel equilibrium problem (2) has a unique solution, see the details in [23].

### Lemma 2.4

([6])

*Let* *C* *be a nonempty closed convex subset of a real Hilbert space* *H* *and* $\phi:C\to\mathbb{R}$ *be a convex*, *lower semicontinuous*, *and subdifferentiable function on* *C*. *Then* $x^{*}$ *is a solution to the convex optimization problem* $\min\{\phi(x): x\in C\}$ *if and only if* $0\in\partial\phi(x^{*})+N_{C}(x^{*})$, *where* $\partial\phi(\cdot)$ *denotes the subdifferential of* *ϕ* *and* $N_{C}(x^{*})$ *is the normal cone of* *C* *at* $x^{*}$.

### Lemma 2.5

([27])

*Let* $\{a_{n}\}$ *be a sequence of nonnegative real numbers*, $\{\alpha_{n}\}$ *be a sequence in* $(0,1)$, *and* $\{\xi _{n}\}$ *be a sequence in* $\mathbb{R}$ *satisfying the condition*

$$a_{n+1}\leq(1-\alpha_{n})a_{n}+\alpha_{n} \xi_{n}\quad \forall n\geq0, $$

*where* $\sum_{n=0}^{\infty}\alpha_{n}=\infty$ *and* $\limsup_{n\rightarrow\infty }\xi_{n}\leq0$. *Then* $\lim_{n\rightarrow\infty}a_{n}=0$.

### Lemma 2.6

([18])

*Let* $\{a_{n}\}$ *be a sequence of real numbers that does not decrease at infinity*, *in the sense that there exists a subsequence* $\{a_{n_{j}}\} $ *of* $\{a_{n}\}$ *such that*

$$a_{n_{j}} < a_{n_{{j}+1}}\quad \textit{for all } j\geq0. $$

*Also consider the sequence of integers* $\{\tau(n)\}_{n\geq n_{0}}$ *defined*, *for all* $n\geq n_{0}$, *by*

$$\tau(n) = \max\{k\leq n| a_{k} < a_{k+1}\}. $$

*Then* $\{\tau(n)\}_{n\geq n_{0}}$ *is a nondecreasing sequence verifying*

$$\lim_{n\rightarrow\infty}\tau(n)=\infty, $$

*and*, *for all* $n\geq n_{0}$, *the following two estimates hold*:

$$a_{\tau(n)}\leq a_{\tau(n)+1} \quad \textit{and}\quad a_{n}\leq a_{\tau(n)+1}. $$

### Lemma 2.7

*Suppose that* *f* *is* *β*-*strongly monotone on* *H* *and satisfies* (A4). *Let* $0<\alpha<1$, $0\leq\eta\leq1-\alpha$, *and* $0< \mu<\frac{2\beta }{L^{2}}$. *For each* $x,y\in H, w\in\partial_{2}f(x,x)$, *and* $v\in\partial_{2}f(y,y)$, *we have*

$$\bigl\Vert (1-\eta)x-\alpha\mu w-\bigl[(1-\eta)y-\alpha\mu v\bigr] \bigr\Vert \leq(1-\eta-\alpha\tau) \Vert x-y \Vert , $$

*where* $\tau=1-\sqrt{1-\mu(2\beta-\mu L^{2})}\in(0,1]$.

### Proof

Let $x,y\in H, w\in\partial_{2}f(x,x)$, and $v\in\partial_{2}f(y,y)$. Thus

4 $$\begin{aligned} & \bigl\Vert (1-\eta)x-\alpha\mu w-\bigl[(1-\eta)y- \alpha\mu v\bigr] \bigr\Vert \\ &\quad \leq \bigl\Vert (1-\eta) (x-y)-\alpha\mu(w-v) \bigr\Vert \\ &\quad = \bigl\Vert (1-\eta-\alpha) (x-y)+\alpha\bigl[(x-y)-\mu(w-v)\bigr] \bigr\Vert \\ &\quad \leq (1-\eta-\alpha) \Vert x-y \Vert +\alpha \bigl\Vert (x-y)- \mu(w-v) \bigr\Vert . \end{aligned}$$

Since $f:H\times H\to\mathbb{R}$ is *β*-strongly monotone, $w\in\partial_{2}f(x,x)$, and $v\in\partial_{2}f(y,y)$, we have

5 $$ -\beta \Vert x-y \Vert ^{2}\geq f(x,y)+f(y,x)\geq- \langle x-y, w-v\rangle. $$

From (5) and (A4), we have

$$\begin{aligned} \bigl\Vert (x-y)-\mu(w-v) \bigr\Vert ^{2} =& \Vert x-y \Vert ^{2}-2\mu\langle x-y,w-v\rangle+\mu ^{2} \Vert w-v \Vert ^{2} \\ \leq& \Vert x-y \Vert ^{2}-2\mu\beta \Vert x-y \Vert ^{2}+\mu^{2}L^{2} \Vert x-y \Vert ^{2} \\ =& \bigl(1-2\mu\beta+\mu^{2}L^{2}\bigr) \Vert x-y \Vert ^{2}. \end{aligned}$$

This implies that

6 $$ \bigl\Vert (x-y)-\mu(w-v) \bigr\Vert \leq\sqrt{1-2\mu\beta+ \mu^{2}L^{2}} \Vert x-y \Vert . $$

By using (4) and (6), we can conclude that

$$\begin{aligned} & \bigl\Vert (1-\eta)x-\alpha\mu w-\bigl[(1-\eta)y-\alpha\mu v \bigr] \bigr\Vert \\ &\quad \leq (1-\eta-\alpha) \Vert x-y \Vert + \bigl(\alpha\sqrt{1-2\mu\beta+ \mu^{2}L^{2}}\bigr) \Vert x-y \Vert \\ &\quad = (1-\eta-\alpha\tau) \Vert x-y \Vert , \end{aligned}$$

where $\tau=1-\sqrt{1-\mu(2\beta-\mu L^{2})}\in(0,1]$. □

## The extragradient subgradient Halpern methods

In this section, we propose the algorithm for finding the solution of a bilevel equilibrium problem under the strong monotonicity of *f* and the pseudomonotonicity and Lipschitz-type continuous conditions on *g*.

### Algorithm 1

Initialization: Choose $x_{0}\in H, 0<\mu< \frac{2\beta }{L^{2}}$, the sequences $\{\alpha_{n}\}\subset(0,1), \{\eta_{n}\}$, and $\{ \lambda_{n}\}$ are such that

$$\textstyle\begin{cases} \lim_{n\rightarrow\infty}\alpha_{n}=0,\qquad \sum_{n=0}^{\infty}\alpha_{n}=\infty,\\ 0\leq\eta_{n}\leq1- \alpha_{n}\quad \forall n\geq0,\qquad \lim_{n\rightarrow\infty}\eta_{n}=\eta< 1,\\ 0< \underline{\lambda}\leq\lambda_{n}\leq\overline{\lambda}< \min ( \frac{1}{2L_{1}},\frac{1}{2L_{2}} ). \end{cases} $$

Set $n=0$ and go to Step 1.

*Step 1*. Compute

$$\begin{aligned} &y_{n}=\operatorname{arg}\min_{y\in C}\biggl\{ \lambda_{n}g(x_{n},y)+\frac{1}{2} \Vert y-x_{n} \Vert ^{2}\biggr\} , \\ &z_{n}=\operatorname{arg}\min_{y\in C}\biggl\{ \lambda_{n}g(y_{n},y)+\frac{1}{2} \Vert y-x_{n} \Vert ^{2}\biggr\} . \end{aligned}$$

*Step 2*. Compute $w_{n}\in\partial_{2}f(z_{n},z_{n})$ and

$$x_{n+1}= \eta_{n}x_{n}+(1-\eta_{n})z_{n}- \alpha_{n}\mu w_{n}. $$

Set $n=n+1$ and go back to Step 1.

### Theorem 3.1

*Let bifunctions* *f* *and* *g* *satisfy Condition *A *and Condition *B, *respectively*. *Assume that* $\varOmega\neq\emptyset$. *Then the sequence* $\{ x_{n}\}$ *generated by Algorithm *1 *converges strongly to the unique solution of the bilevel equilibrium problem* (2).

### Proof

Under assumptions of two bifunctions *f* and *g*, we get the unique solution of the bilevel equilibrium problem (2), denoted by $x^{*}$.

*Step 1*: Show that

7 $$ \bigl\Vert z_{n}-x^{*} \bigr\Vert ^{2}\leq \bigl\Vert x_{n}-x^{*} \bigr\Vert ^{2}-(1-2\lambda_{n}L_{1}) \Vert x_{n}-y_{n} \Vert ^{2}-(1-2\lambda_{n}L_{2}) \Vert y_{n}-z_{n} \Vert ^{2}. $$

The definition of $y_{n}$ and Lemma 2.4 imply that

$$0\in\partial_{2} \biggl\{ \lambda_{n}g(x_{n},y)+ \frac{1}{2} \Vert y-x_{n} \Vert ^{2} \biggr\} (y_{n})+N_{C}(y_{n}). $$

There are $w\in\partial_{2}g(x_{n},y_{n})$ and $\bar{w}\in N_{C}(y_{n})$ such that

8 $$ \lambda_{n}w+y_{n}-x_{n}+\bar{w}=0. $$

Since $\bar{w}\in N_{C}(y_{n})$, we have

9 $$ \langle\bar{w},y-y_{n}\rangle\leq0\quad \text{for all } y\in C. $$

By using (8) and (9), we obtain $\lambda_{n}\langle w,y-y_{n}\rangle\geq\langle x_{n}-y_{n},y-y_{n}\rangle$ for all $y\in C$. Since $z_{n}\in C$, we have

10 $$ \lambda_{n}\langle w,z_{n}-y_{n} \rangle\geq\langle x_{n}-y_{n},z_{n}-y_{n} \rangle. $$

It follows from $w\in\partial_{2}g(x_{n},y_{n})$ that

11 $$ g(x_{n},y)-g(x_{n},y_{n})\geq\langle w, y-y_{n}\rangle\quad \text{for all } y\in H. $$

By using (10) and (11), we get

12 $$ \lambda_{n} \bigl\{ g(x_{n},z_{n})-g(x_{n},y_{n}) \bigr\} \geq\langle x_{n}-y_{n},z_{n}-y_{n} \rangle. $$

Similarly, the definition of $z_{n}$ implies that

$$0\in\partial_{2} \biggl\{ \lambda_{n}g(y_{n},y)+ \frac{1}{2} \Vert y-x_{n} \Vert ^{2} \biggr\} (z_{n})+N_{C}(z_{n}). $$

There are $u\in\partial_{2}g(y_{n},z_{n})$ and $\bar{u}\in N_{C}(z_{n})$ such that

13 $$ \lambda_{n}u+z_{n}-x_{n}+\bar{u}=0. $$

Since $\bar{u}\in N_{C}(z_{n})$, we have

14 $$ \langle\bar{u},y-z_{n}\rangle\leq0\quad \text{for all } y\in C. $$

By using (13) and (14), we obtain $\lambda_{n}\langle u,y-z_{n}\rangle\geq\langle x_{n}-z_{n},y-z_{n}\rangle$ for all $y\in C$. Since $x^{*}\in C$, we have

15 $$ \lambda_{n}\bigl\langle u,x^{*}-z_{n} \bigr\rangle \geq\bigl\langle x_{n}-z_{n},x^{*}-z_{n} \bigr\rangle . $$

It follows from $u\in\partial_{2}g(y_{n},z_{n})$ that

16 $$ g(y_{n},y)-g(y_{n},z_{n})\geq\langle u, y-z_{n}\rangle\quad \text{for all } y\in H. $$

By using (15) and (16), we get

17 $$ \lambda_{n} \bigl\{ g\bigl(y_{n},x^{*} \bigr)-g(y_{n},z_{n}) \bigr\} \geq\bigl\langle x_{n}-z_{n},x^{*}-z_{n}\bigr\rangle . $$

Since $x^{*}\in\varOmega$, we have $g(x^{*},y_{n})\geq0$. If follows from the pseudomonotonicity of *g* on *C* with respect to *Ω* that $g(y_{n},x^{*})\leq0$. This implies that

18 $$ \bigl\langle x_{n}-z_{n},z_{n}-x^{*} \bigr\rangle \geq\lambda_{n}g(y_{n},z_{n}). $$

Since *g* is Lipschitz-type continuous, there exist two positive constants $L_{1},L_{2}$ such that

19 $$ g(y_{n},z_{n})\geq g(x_{n},z_{n})- g(x_{n},y_{n})- L_{1} \Vert x_{n}-y_{n} \Vert ^{2}-L_{2} \Vert y_{n}-z_{n} \Vert ^{2}. $$

By using (18) and (19), we get

$$\bigl\langle x_{n}-z_{n},z_{n}-x^{*} \bigr\rangle \geq\lambda_{n}\bigl\{ g(x_{n},z_{n})- g(x_{n},y_{n})\bigr\} -\lambda_{n}L_{1} \Vert x_{n}-y_{n} \Vert ^{2}- \lambda_{n}L_{2} \Vert y_{n}-z_{n} \Vert ^{2}. $$

From (12) and the above inequality, we obtain

20 $$ 2\bigl\langle x_{n}-z_{n},z_{n}-x^{*} \bigr\rangle \geq2\langle x_{n}-y_{n},z_{n}-y_{n} \rangle -\lambda_{n}L_{1} \Vert x_{n}-y_{n} \Vert ^{2}-\lambda_{n}L_{2} \Vert y_{n}-z_{n} \Vert ^{2}. $$

We know that

$$\begin{aligned} &2\bigl\langle x_{n}-z_{n},z_{n}-x^{*} \bigr\rangle = \bigl\Vert x_{n}-x^{*} \bigr\Vert ^{2}- \Vert z_{n}-x_{n} \Vert ^{2}- \bigl\Vert z_{n}-x^{*} \bigr\Vert ^{2}, \\ &2\langle x_{n}-y_{n},z_{n}-y_{n} \rangle= \Vert x_{n}-y_{n} \Vert ^{2}+ \Vert z_{n}-y_{n} \Vert ^{2}- \Vert x_{n}-z_{n} \Vert ^{2}. \end{aligned}$$

From (20), we can conclude that

$$\bigl\Vert z_{n}-x^{*} \bigr\Vert ^{2}\leq \bigl\Vert x_{n}-x^{*} \bigr\Vert ^{2}-(1-2 \lambda_{n}L_{1}) \Vert x_{n}-y_{n} \Vert ^{2} -(1-2\lambda_{n}L_{2}) \Vert y_{n}-z_{n} \Vert ^{2}. $$

*Step 2*: The sequences $\{x_{n}\}, \{w_{n}\}, \{ y_{n}\}$, and $\{z_{n}\}$ are bounded.

Since $0<\lambda_{n}<a$, where $a=\min ( \frac {1}{2L_{1}},\frac{1}{2L_{2}} )$, we have

$$(1-2\lambda_{n}L_{1})>0\quad \text{and}\quad (1-2 \lambda_{n}L_{2})>0. $$

It follows from (7) and the above inequalities that

21 $$ \bigl\Vert z_{n}-x^{*} \bigr\Vert \leq \bigl\Vert x_{n}-x^{*} \bigr\Vert \quad \text{for all } n \in \mathbb{N}. $$

By Lemma 2.7 and (21), we obtain

22 $$\begin{aligned} \bigl\Vert x_{n+1}-x^{*} \bigr\Vert =& \bigl\Vert \eta_{n}x_{n}+(1- \eta_{n})z_{n}-\alpha_{n}\mu w_{n} -x^{*}+\eta_{n}x^{*}-\eta_{n}x^{*}+ \alpha_{n}\mu v-\alpha_{n}\mu v \bigr\Vert \\ =& \bigl\Vert (1-\eta_{n})z_{n}- \alpha_{n}\mu w_{n}-(1-\eta_{n})x^{*}+ \alpha _{n}\mu v +\eta\bigl(x_{n}-x^{*}\bigr)- \alpha_{n}\mu v \bigr\Vert \\ \leq& \bigl\Vert (1-\eta_{n})z_{n}- \alpha_{n}\mu w_{n}-\bigl[(1-\eta _{n})x^{*}- \alpha_{n}\mu v\bigr] \bigr\Vert +\eta_{n} \bigl\Vert x_{n}-x^{*} \bigr\Vert + \alpha_{n}\mu \Vert v \Vert \\ \leq& (1-\eta_{n}-\alpha_{n}\tau) \bigl\Vert z_{n}-x^{*} \bigr\Vert +\eta_{n} \bigl\Vert x_{n}-x^{*} \bigr\Vert + \alpha_{n}\mu \Vert v \Vert \\ \leq& (1-\eta_{n}-\alpha_{n}\tau) \bigl\Vert x_{n}-x^{*} \bigr\Vert +\eta_{n} \bigl\Vert x_{n}-x^{*} \bigr\Vert + \alpha_{n}\mu \Vert v \Vert \\ =& (1-\alpha_{n}\tau) \bigl\Vert x_{n}-x^{*} \bigr\Vert + \alpha_{n}\mu \Vert v \Vert \\ =& (1-\alpha_{n}\tau) \bigl\Vert x_{n}-x^{*} \bigr\Vert + \alpha_{n}\tau \biggl(\frac{\mu \Vert v \Vert }{\tau} \biggr), \end{aligned}$$

where $w_{n}\in\partial_{2}f(z_{n},z_{n})$ and $v\in\partial _{2}f(x^{*},x^{*})$. This implies that

$$\bigl\Vert x_{n+1}-x^{*} \bigr\Vert \leq\max \biggl\{ \bigl\Vert x_{n}-x^{*} \bigr\Vert ,\frac{\mu \Vert v \Vert }{\tau } \biggr\} . $$

By induction, we obtain

$$\bigl\Vert x_{n}-x^{*} \bigr\Vert \leq\max \biggl\{ \bigl\Vert x_{0}-x^{*} \bigr\Vert ,\frac{\mu \Vert v \Vert }{\tau } \biggr\} . $$

Thus the sequence $\{x_{n}\}$ is bounded. By using (21), we have $\{z_{n}\}$, and using Condition (A5), we can conclude that $\{w_{n}\}$ is also bounded.

*Step 3*: Show that the sequence $\{x_{n}\}$ converges strongly to $x^{*}$.

Since $x\in\varOmega^{*}$, we have $f(x^{*},y)\geq0$ for all $y\in\varOmega$. Thus $x^{*}$ is a minimum of the convex function $f(x^{*},\cdot)$ over *Ω*. By Lemma 2.4, we obtain $0\in\partial_{2}f(x^{*},x^{*})+N_{\varOmega}(x^{*})$. Then there exists $v\in\partial_{2}f(x^{*},x^{*})$ such that

23 $$ \bigl\langle v, z-x^{*}\bigr\rangle \geq0\quad \text{for all } z\in\varOmega. $$

Note that

24 $$ \Vert x-y \Vert ^{2}\leq \Vert x \Vert ^{2}-2\langle y,x-y\rangle\quad \text{for all } x,y\in H. $$

From Lemma (2.7) and (24), we obtain

$$\begin{aligned} \bigl\Vert x_{n+1}-x^{*} \bigr\Vert ^{2} =& \bigl\Vert \eta_{n}x_{n}+(1-\eta_{n})z_{n} -\alpha_{n}\mu w_{n}-x^{*} \bigr\Vert ^{2} \\ =& \bigl\Vert (1-\eta_{n})z_{n}-\alpha_{n}\mu w_{n}-\bigl[(1-\eta_{n})x^{*}-\alpha _{n} \mu v\bigr] \\ & {}+\eta_{n}\bigl(x_{n}-x^{*}\bigr)- \alpha_{n}\mu v \bigr\Vert ^{2} \\ \leq& \bigl\Vert (1-\eta_{n})z_{n}-\alpha_{n} \mu w_{n}-\bigl[(1-\eta _{n})x^{*}- \alpha_{n}\mu v\bigr] \\ & {}+\eta_{n}\bigl(x_{n}-x^{*}\bigr) \bigr\Vert ^{2}-2\alpha_{n}\mu\bigl\langle v,x_{n+1}-x^{*} \bigr\rangle \\ \leq& \bigl\{ \bigl\Vert (1-\eta_{n})z_{n}- \alpha_{n}\mu w_{n}-\bigl[(1-\eta _{n})x^{*}- \alpha_{n}\mu v\bigr] \\ &{} +\eta_{n}\|\bigl(x_{n}-x^{*}\bigr) \bigr\Vert \bigr\} ^{2}-2\alpha_{n}\mu\bigl\langle v,x_{n+1}-x^{*}\bigr\rangle \\ \leq& \bigl[(1-\eta_{n}-\alpha_{n}\tau) \bigl\Vert z_{n}-x^{*} \bigr\Vert +\eta_{n} \bigl\Vert x_{n}-x^{*} \bigr\Vert \bigr]^{2} -2 \alpha_{n}\mu\bigl\langle v,x_{n+1}-x^{*}\bigr\rangle \\ \leq& (1-\eta_{n}-\alpha_{n}\tau) \bigl\Vert z_{n}-x^{*} \bigr\Vert ^{2}+\eta_{n} \bigl\Vert x_{n}-x^{*} \bigr\Vert ^{2} -2 \alpha_{n}\mu\bigl\langle v,x_{n+1}-x^{*}\bigr\rangle \\ \leq& (1-\eta_{n}-\alpha_{n}\tau) \bigl\Vert x_{n}-x^{*} \bigr\Vert ^{2}+\eta_{n} \bigl\Vert x_{n}-x^{*} \bigr\Vert ^{2} -2 \alpha_{n}\mu\bigl\langle v,x_{n+1}-x^{*}\bigr\rangle \\ =& (1-\alpha_{n}\tau) \bigl\Vert x_{n}-x^{*} \bigr\Vert ^{2}+2\alpha_{n}\mu\bigl\langle v,x^{*}-x_{n+1}\bigr\rangle . \end{aligned}$$

It follows that

25 $$ \bigl\Vert x_{n+1}-x^{*} \bigr\Vert ^{2} \leq(1-\alpha_{n}\tau) \bigl\Vert x_{n}-x^{*} \bigr\Vert ^{2}+2\alpha_{n}\mu\bigl\langle v,x^{*}-x_{n+1}\bigr\rangle . $$

Let us consider two cases.

Case 1: There exists $n_{0}$ such that $\{\|x_{n}-x^{*}\|\}$ is decreasing for $n\geq n_{0}$. Therefore the limit of sequence $\{\|x_{n}-x^{*}\|\} $ exists. By using (21) and (25), we obtain

26 $$\begin{aligned} 0 \leq& \bigl\Vert x_{n}-x^{*} \bigr\Vert ^{2}- \bigl\Vert z_{n}-x^{*} \bigr\Vert ^{2} \\ \leq& - \frac{\alpha_{n}\tau}{1-\eta_{n}} \bigl\Vert z_{n}-x^{*} \bigr\Vert ^{2}-\frac{2\alpha _{n}\mu}{1-\eta_{n}} \bigl\langle v,x_{n+1}-x^{*} \bigr\rangle \\ &{} +\frac{1}{1-\eta_{n}}\bigl( \bigl\Vert x_{n}-x^{*} \bigr\Vert ^{2}- \bigl\Vert x_{n+1}-x^{*} \bigr\Vert ^{2}\bigr) . \end{aligned}$$

Since $\lim_{n\rightarrow\infty}\eta_{n}=\eta<1, \lim_{n\rightarrow \infty}\alpha_{n}=0$ and the limit of $\{\|x_{n}-x^{*}\|\}$ exists, we have

27 $$ \lim_{n\rightarrow\infty}\bigl( \bigl\Vert x_{n}-x^{*} \bigr\Vert ^{2}- \bigl\Vert z_{n}-x^{*} \bigr\Vert ^{2}\bigr)=0. $$

From $0< \lambda_{n}<a$ and inequality (7), we get

$$(1-2a) \Vert x_{n}-y_{n} \Vert ^{2} \leq(1-2 \lambda_{n}L_{1}) \Vert x_{n}-y_{n} \Vert ^{2} \leq \bigl\Vert x_{n}-x^{*} \bigr\Vert ^{2}- \bigl\Vert z_{n}-x^{*} \bigr\Vert ^{2}. $$

By using (27), we obtain $\lim_{n\rightarrow\infty}\|x_{n}-y_{n}\|=0$. Next, we show that

28 $$ \limsup_{n\rightarrow\infty}\bigl\langle v,x^{*}-x_{n+1} \bigr\rangle \leq0. $$

Take a subsequence $\{x_{n_{k}}\}$ of $\{x_{n}\}$ such that

$$\limsup_{n\rightarrow\infty}\bigl\langle v,x^{*}-x_{n+1} \bigr\rangle = \limsup_{k\rightarrow\infty}\bigl\langle v,x^{*}-x_{n_{k}} \bigr\rangle . $$

Since $\{x_{n_{k}}\}$ is bounded, we may assume that $\{x_{n_{k}}\}$ converges weakly to some $\bar{x}\in H$. Therefore

29 $$ \limsup_{n\rightarrow\infty}\bigl\langle v,x^{*}-x_{n+1} \bigr\rangle = \limsup_{k\rightarrow\infty}\bigl\langle v,x^{*}-x_{n_{k}} \bigr\rangle =\bigl\langle v,x^{*}-\bar{x}\bigr\rangle . $$

Since $\lim_{n\rightarrow\infty}\|x_{n}-y_{n}\|=0$ and $x_{n_{k}}\rightharpoonup\bar{x}$, we have $y_{n_{k}}\rightharpoonup \bar{x}$. Since *C* is closed and convex, it is also weakly closed and thus $\bar {x}\in C$. Next, we show that $\bar{x}\in\varOmega$. From the definition of $\{y_{n}\}$ and Lemma 2.4, we obtain

$$0\in\partial_{2}\biggl\{ \lambda_{n}g(x_{n},y)+ \frac{1}{2} \Vert x_{n}-y_{n} \Vert ^{2} \biggr\} (y_{n}) +N_{C}(y_{n}). $$

There exist $\bar{w}\in N_{C}(y_{n})$ and $w\in\partial _{2}g(x_{n},y_{n})$ such that

30 $$ \lambda_{n}w+y_{n}-x_{n}+\bar{w}=0. $$

Since $\bar{w}\in N_{C}(y_{n})$, we have $\langle\bar{w},y- y_{n}\rangle\leq0$ for all $y\in C$. From (30), we obtain

31 $$ \lambda_{n}\langle w,y- y_{n}\rangle\geq \langle x_{n}-y_{n},y- y_{n}\rangle \quad \text{for all } y\in C. $$

Since $w\in\partial_{2}g(x_{n},y_{n})$, we have

32 $$ g(x_{n},y)-g(x_{n},y_{n})\geq\langle w,y- y_{n}\rangle\quad \text{for all }y\in H. $$

Combining (31) and (32), we get

33 $$ \lambda_{n}\bigl\{ g(x_{n},y)-g(x_{n},y_{n}) \bigr\} \geq\langle x_{n}-y_{n},y- y_{n}\rangle\quad \text{for all } y\in C. $$

Taking $n=n_{k}$ and $k\rightarrow\infty$ in (33), the assumption of $\lambda_{n}$ and (B5), we obtain $g(\bar{x},y)\geq0$ for all $y\in C$. This implies that $\bar{x}\in\varOmega$. By inequality (23), we obtain $\langle v, \bar{x}- x^{*}\rangle \geq0$. It follows from (29) that

34 $$ \limsup_{n\rightarrow\infty}\bigl\langle v,x^{*}-x_{n+1} \bigr\rangle \leq0. $$

We can write inequality (25) in the following form:

$$\bigl\Vert x_{n+1}-x^{*} \bigr\Vert ^{2}\leq(1- \alpha_{n}\tau) \bigl\Vert x_{n}-x^{*} \bigr\Vert ^{2}-\alpha _{n}\tau\xi_{n}, $$

where $\xi_{n}=\frac{2\mu}{\tau}\langle v,x^{*}-x_{n+1}\rangle$. It follows from (34) that $\limsup_{n\rightarrow\infty}\xi _{n}\leq0$. By Lemma 2.5, we can conclude that $\lim_{n\rightarrow\infty}\|x_{n}-x^{*}\|^{2}=0$. Hence $x_{n}\rightarrow x^{*}$ as $n \rightarrow\infty$.

Case 2: There exists a subsequence $\{x_{n_{j}}\}$ of $\{x_{n}\} $ such that $\|x_{n_{j}}-x^{*}\|\leq\|x_{n_{j}+1}-x^{*}\|$ for all $j\in\mathbb{N}$. By Lemma 2.6, there exists a nondecreasing sequence $\{\tau (n)\}$ of $\mathbb{N}$ such that $\lim_{n\rightarrow\infty}\tau(n)=\infty$, and for each sufficiently large $n\in\mathbb{N}$, we have

35 $$ \bigl\Vert x_{\tau(n)}-x^{*} \bigr\Vert \leq \bigl\Vert x_{\tau(n)+1}-x^{*} \bigr\Vert \quad \text{and}\quad \bigl\Vert x_{n}-x^{*} \bigr\Vert \leq \bigl\Vert x_{\tau(n)+1}-x^{*} \bigr\Vert . $$

Combining (22) and (35), we have

36 $$\begin{aligned} \bigl\Vert x_{\tau(n)}-x^{*} \bigr\Vert \leq& \bigl\Vert x_{\tau(n)+1}-x^{*} \bigr\Vert \\ \leq& (1-\eta_{\tau(n)}-\alpha_{\tau(n)}\tau) \Vert z_{\tau(n)-x^{*}} \Vert +\eta_{\tau(n)} \bigl\Vert x_{\tau(n)}-x^{*} \bigr\Vert +\alpha_{\tau(n)}\mu \Vert v \Vert . \end{aligned}$$

From (21) and (36), we get

37 $$ 0 \leq \bigl\Vert x_{\tau(n)}-x^{*} \bigr\Vert - \bigl\Vert z_{\tau(n)}-x^{*} \bigr\Vert \leq- \frac{\alpha_{\tau(n)}\tau}{1-\eta_{\tau(n)}} \bigl\Vert z_{\tau (n)}-x^{*} \bigr\Vert + \frac{\alpha_{\tau(n)}\mu}{1-\eta_{\tau(n)}} \Vert v \Vert . $$

Since $\lim_{n\rightarrow\infty}\alpha_{n}=0$, $\lim_{n\rightarrow\infty }\eta_{n}=\eta<1$, $\{z_{n}\}$ is bounded, and (37), we have $\lim_{n\rightarrow\infty}(\|x_{\tau(n)}-x^{*}\|-\|z_{\tau (n)}-x^{*}\|)=0$. It follows from the boundedness of $\{x_{n}\}$ and $\{z_{n}\}$ that

38 $$ \lim_{n\rightarrow\infty}\bigl( \bigl\Vert x_{\tau(n)}-x^{*} \bigr\Vert ^{2}- \bigl\Vert z_{\tau (n)}-x^{*} \bigr\Vert ^{2}\bigr)=0. $$

By using the assumption of $\{\lambda_{n}\}$, we get the following two inequalities:

$$1-2\lambda_{\tau(n)}L_{1}>1-2aL_{1}>0 \quad \text{and}\quad 1-2\lambda_{\tau(n)}L_{2}>1-2aL_{2}>0. $$

From (7), we obtain

$$\begin{aligned} \bigl\Vert z_{\tau(n)}-x^{*} \bigr\Vert ^{2} \leq& \bigl\Vert x_{\tau(n)}-x^{*} \bigr\Vert ^{2}-(1-2\lambda _{\tau(n)}L_{1}) \Vert x_{\tau(n)}-y_{\tau(n)} \Vert ^{2} \\ &{} -(1-2\lambda_{\tau(n)}L_{2}) \Vert y_{\tau(n)}-z_{\tau(n)} \Vert ^{2} \\ \leq& \bigl\Vert x_{\tau(n)}-x^{*} \bigr\Vert ^{2}-(1-2aL_{1}) \Vert x_{\tau(n)}-y_{\tau(n)} \Vert ^{2} \\ &{}-(1-2aL_{2}) \Vert y_{\tau(n)}-z_{\tau(n)} \Vert ^{2}. \end{aligned}$$

This implies that

$$\begin{aligned} 0&< (1-2aL_{1}) \Vert x_{\tau(n)}-y_{\tau(n)} \Vert ^{2} +(1-2aL_{2}) \Vert y_{\tau(n)}-z_{\tau(n)} \Vert ^{2}\\ &\leq \bigl\Vert x_{\tau(n)}-x^{*} \bigr\Vert ^{2}- \bigl\Vert z_{\tau(n)}-x^{*} \bigr\Vert ^{2}. \end{aligned}$$

It follows from (38) and the above inequality that

39 $$ \lim_{n\rightarrow\infty} \Vert x_{\tau(n)}-y_{\tau(n)} \Vert =0\quad \text{and}\quad \lim_{n\rightarrow\infty} \Vert y_{\tau (n)}-z_{\tau(n)} \Vert =0. $$

Note that $\|x_{\tau(n)}-z_{\tau(n)}\|\leq\|x_{\tau(n)}-y_{\tau(n)}\|+\|y_{\tau (n)}-z_{\tau(n)}\|$. From (39), we have

40 $$ \lim_{n\rightarrow\infty} \Vert x_{\tau(n)}-z_{\tau(n)} \Vert =0. $$

By using the definition of $x_{n+1}$ and Lemma 2.7, we obtain

$$\begin{aligned} \Vert x_{\tau(n)+1}-x_{\tau(n)} \Vert =& \bigl\Vert \eta_{\tau(n)}x_{\tau(n)}+(1-\eta _{\tau(n)})z_{\tau(n)} - \alpha_{\tau(n)}\mu t_{\tau(n)}-x_{\tau(n)} \bigr\Vert \\ =& \bigl\Vert (1-\eta_{\tau(n)})z_{\tau(n)}-\alpha_{\tau(n)} \mu t_{\tau(n)} \\ &{} -\bigl[(1-\eta_{\tau(n)})x_{\tau(n)}-\alpha_{\tau(n)}w_{\tau(n)} \bigr]-\alpha _{\tau(n)}w_{\tau(n)} \bigr\Vert \\ \leq& \bigl\Vert (1-\eta_{\tau(n)})z_{\tau(n)}- \alpha_{\tau(n)}t_{\tau(n)} \\ &{} -\bigl[(1-\eta_{\tau(n)})x_{\tau(n)}-\alpha_{\tau(n)}w_{\tau(n)} \bigr] \bigr\Vert +\alpha_{\tau(n)} \Vert w_{\tau(n)} \Vert \\ \leq& (1-\eta_{\tau(n)}-\alpha_{\tau(n)}\tau) \Vert z_{\tau(n)}-x_{\tau (n)} \Vert +\alpha_{\tau(n)} \Vert w_{\tau(n)} \Vert \\ \leq& \Vert z_{\tau(n)}-x_{\tau(n)} \Vert +\alpha_{\tau(n)} \Vert w_{\tau(n)} \Vert , \end{aligned}$$

where $t_{\tau(n)}\in\partial_{2}f(z_{\tau(n)},z_{\tau(n)})$ and $w_{\tau(n)}\in\partial_{2}f(x_{\tau(n)},x_{\tau(n)})$. Since $\lim_{n\rightarrow\infty}\alpha_{n}=0$, the boundedness of $\{ w_{\tau(n)}\}$ and (40), we have $\lim_{n\rightarrow\infty}\|x_{\tau(n)+1}-x_{\tau(n)}\|=0$. As proved in the first case, we can conclude that

41 $$ \limsup_{n\rightarrow\infty}\bigl\langle v,x^{*}-x_{\tau(n)+1} \bigr\rangle = \limsup_{k\rightarrow\infty}\bigl\langle v,x^{*}-x_{\tau(n)} \bigr\rangle \leq0. $$

Combining (25) and (35), we obtain

$$\begin{aligned} \bigl\Vert x_{\tau(n)+1}-x^{*} \bigr\Vert ^{2} \leq& (1-\alpha_{\tau(n)}\tau) \bigl\Vert x_{\tau (n)}-x^{*} \bigr\Vert ^{2} +2 \alpha_{\tau(n)}\mu\bigl\langle v,x^{*}-x_{\tau(n)+1}\bigr\rangle \\ \leq& (1-\alpha_{\tau(n)}\tau) \bigl\Vert x_{\tau(n)+1}-x^{*} \bigr\Vert ^{2} +2\alpha_{\tau(n)}\mu\bigl\langle v,x^{*}-x_{\tau(n)+1}\bigr\rangle . \end{aligned}$$

By using (35) again, we have

42 $$ \bigl\Vert x_{n}-x^{*} \bigr\Vert ^{2}\leq \bigl\Vert x_{\tau(n)+1}-x^{*} \bigr\Vert ^{2}\leq\frac{2\mu}{\tau }\bigl\langle v,x^{*}-x_{\tau(n)+1} \bigr\rangle . $$

From (41), we can conclude that $\limsup_{n\rightarrow\infty}\|x_{n}-x^{*}\|^{2}\leq0$. Hence $x_{n}\rightarrow x^{*}$ as $n\rightarrow\infty$. This completes the proof. □

## The extragradient subgradient methods with line searches

In this section, we introduce the algorithm for finding the solution of a bilevel equilibrium problem without the Lipschitz condition for the bifunction *g*.

### Algorithm 2

Initialization: Choose $x_{0}\in C$, $0<\mu< \frac{2\beta }{L^{2}}$, $\rho\in(0,2)$, $\gamma\in(0,1)$, the sequences $\{\lambda_{n}\}$, $\{\xi_{n}\}$, and $\{\alpha_{n}\}\subset(0,1)$ such that

$$\textstyle\begin{cases} \lim_{n\rightarrow\infty}\alpha_{n}=0,\qquad \sum_{n=0}^{\infty}\alpha_{n}=\infty, \qquad \sum_{n=0}^{\infty}\alpha_{n}^{2}< \infty,\\ \lambda_{n}\in[\underline{\lambda},\overline{\lambda}] \subset (0,\infty),\qquad \xi_{n}\in[\underline{\xi},\overline{\xi }]\subset(0,2). \end{cases} $$

Set $n=0$, and go to Step 2.

*Step 1*. Compute

$$y_{n}=\operatorname{arg}\min_{y\in C}\biggl\{ \lambda_{n}g(x_{n},y)+\frac{1}{2} \Vert y-x_{n} \Vert ^{2}\biggr\} . $$

If $y_{n}=x_{n}$, then set $u_{n}=x_{n}$ and go to Step 4. Otherwise, go to Step 2.

*Step 2*. (Armijo line search rule) Find *m* as the smallest positive integer number satisfying

$$\textstyle\begin{cases} z_{n,m}=(1-\gamma^{m})x_{n}+\gamma^{m}y_{n}, \\ g(z_{n,m}, x_{n})-g(z_{n,m},y_{n})\geq\frac{\rho}{2\lambda_{n}} \Vert x_{n}-y_{n} \Vert ^{2}. \end{cases} $$

Set $z_{n}=z_{n,m}$ and $\gamma_{n}=\gamma^{m}$.

*Step 3*. Choose $t_{n}\in\partial_{2}g(z_{n},x_{n})$ and compute $u_{n}=P_{C}(x_{n}-\xi_{n}\sigma_{n}t_{n})$ where $\sigma _{n}=\frac{g(z_{n},x_{n})}{\|t_{n}\|^{2}}$

*Step 4*. Compute $w_{n}\in\partial_{2}f(u_{n},u_{n})$ and

$$x_{n+1}= P_{C}(u_{n}-\alpha_{n}\mu w_{n}). $$

Set $n=n+1$, and go back to Step 1.

### Lemma 4.1

([26])

*Suppose that* $y_{n}\neq x_{n}$ *for some* $n \in\mathbb{N}$. *Then the line search corresponding to* $x_{n}$ *and* $y_{n}$ (*Step* 2) *is well defined*, $g(z_{n}, x_{n}) > 0$, *and* $0 \notin\partial_{2}g(z_{n},x_{n})$.

### Lemma 4.2

([26])

*Let* $g:\varTheta\times\varTheta \rightarrow\infty$ *be a bifunction satisfying conditions* (B1) *on* *C* *and* (B5) *on* *Θ*, *where* *Θ* *is an open convex set containing* *C*. *Let* $\bar{x}, \bar{y}\in\varTheta$ *and* $\{x_{n}\}, \{y_{n}\}$ *be two sequences in* *Θ* *converging weakly to* $\bar{x}, \bar{y}\in\varTheta$, *respectively*. *Then*, *for any* $\varepsilon>0$, *there exist* $\eta>0$ *and* $n_{\varepsilon}\in\mathbb{N}$ *such that*

$$\partial_{2}g(x_{n},y_{n})\subset \partial_{2}g(\bar{x}, \bar{y})+\frac {\varepsilon}{\eta}B $$

*for all* $n\geq n_{\varepsilon}$, *where* *B* *denotes the closed unit ball in* *H*.

### Lemma 4.3

([8])

*Let the bifunction* *g* *satisfy assumption* (B1) *on* $C\times C$, (B5) *on* $\varTheta\times\varTheta$. *Suppose that* $\{x_{n}\}$ *is a bounded sequence in* $C, \rho>0$ *and* $\{y_{n}\}$ *is a sequence such that*

$$y_{n}=\operatorname{arg}\min_{y\in C}\biggl\{ g(x_{n},y)+\frac{\rho}{2} \Vert y-x_{n} \Vert ^{2}\biggr\} . $$

*Then* $\{y_{n}\}$ *is also bounded*.

### Theorem 4.4

*Let the bifunction* *f* *satisfy Condition *A *and* *g* *satisfy Conditions* (B1)–(B3) *and* (B5). *Assume that* $\varOmega\neq\emptyset$. *Then the sequences* $\{x_{n}\}$ *generated by Algorithm* 2 *converge strongly to the unique solution of the bilevel equilibrium problem* (2).

### Proof

Let $x^{*}$ be the unique solution of the bilevel equilibrium problem (2). Then we have $x^{*}\in\varOmega$ and there exists $v\in\partial _{2}f(x^{*},x^{*})$ such that

43 $$ \bigl\langle v, z-x^{*}\bigr\rangle \geq0\quad \text{for all } z\in\varOmega. $$

*Step 1*: Show that

44 $$ \bigl\Vert u_{n}-x^{*} \bigr\Vert ^{2}\leq \bigl\Vert x_{n}-x^{*} \bigr\Vert ^{2}-\xi_{n}(2-\xi_{2}) \bigl(\sigma _{n} \Vert t_{n} \Vert \bigr)^{2}. $$

By the definition of $u_{n}$, we have

45 $$\begin{aligned} \bigl\Vert u_{n}-x^{*} \bigr\Vert ^{2} \leq& \bigl\Vert P_{C}(x_{n}- \xi_{n}\sigma _{n}t_{n})-P_{C} \bigl(x^{*}\bigr) \bigr\Vert ^{2} \\ \leq& \bigl\Vert x_{n}-\xi_{n} \sigma_{n}t_{n}-x^{*} \bigr\Vert ^{2} \\ =& \bigl\Vert x_{n}-x^{*} \bigr\Vert ^{2}-2 \xi_{n}\sigma_{n}\bigl\langle t_{n},x_{n}-x^{*} \bigr\rangle +\bigl(\xi_{n}\sigma_{n} \Vert t_{n} \Vert \bigr)^{2}. \end{aligned}$$

Since $t_{n}\in\partial_{2}g(z_{n},x_{n})$ and $g(z_{n},\cdot)$ is convex on *C*, we have $g(z_{n},x^{*})-g(z_{n},x_{n})\geq\langle t_{n}, x^{*}-x_{n}\rangle$. It follows that

46 $$ \bigl\langle t_{n},x_{n}-x^{*}\bigr\rangle \geq g(z_{n},x_{n})-g\bigl(z_{n},x^{*} \bigr). $$

Since *g* is pseudomonotone on *C* with respect to *Ω*, we have $g(z_{n},x^{*})\leq0$. It follows from (46) and the definition of $\sigma_{n}$ that

47 $$ \bigl\langle t_{n},x_{n}-x^{*}\bigr\rangle \geq g(z_{n},x_{n})=\sigma_{n} \Vert t_{n} \Vert ^{2}. $$

Combining (45) with (47), we obtain

$$\begin{aligned} \bigl\Vert u_{n}-x^{*} \bigr\Vert ^{2} \leq& \bigl\Vert x_{n}-x^{*} \bigr\Vert ^{2}-2\xi_{n}\sigma _{n}\bigl(\sigma_{n} \Vert t_{n} \Vert ^{2}\bigr) +\bigl(\xi_{n} \sigma_{n} \Vert t_{n} \Vert \bigr)^{2} \\ =& \bigl\Vert x_{n}-x^{*} \bigr\Vert ^{2}-2 \xi_{n}\bigl(\sigma_{n} \Vert t_{n} \Vert \bigr)^{2} +{\xi_{n}}^{2}\bigl(\sigma_{n} \Vert t_{n} \Vert \bigr)^{2} \\ =& \bigl\Vert x_{n}-x^{*} \bigr\Vert ^{2}- \xi_{n}(2-\xi_{n}) \bigl(\sigma_{n} \Vert t_{n} \Vert \bigr)^{2}. \end{aligned}$$

*Step 2*: The sequences $\{x_{n}\}$, $\{y_{n}\}$, $\{u_{n}\}$, and $\{w_{n}\}$ are bounded.

Since $\xi_{n}\in[\underline{\xi},\overline{\xi}]\subset (0,2)$ and (44), we have

48 $$ \bigl\Vert u_{n}-x^{*} \bigr\Vert \leq \bigl\Vert x_{n}-x^{*} \bigr\Vert . $$

By the definition of $x_{n+1}$, we get

49 $$\begin{aligned} \bigl\Vert x_{n+1}-x^{*} \bigr\Vert =& \bigl\Vert P_{C}(u_{n}- \alpha_{n}\mu w_{n})-P_{C}\bigl(x^{*} \bigr) \bigr\Vert \\ \leq& \bigl\Vert u_{n}-\alpha_{n}\mu w_{n}-x^{*} \bigr\Vert \\ \leq& \bigl\Vert \bigl(u_{n}-x^{*}\bigr)- \alpha_{n}\mu(w_{n}-v)-\alpha_{n}\mu v \bigr\Vert \\ =& \bigl\Vert \bigl(u_{n}- x^{*}\bigr)- \alpha_{n}\mu(w_{n}-v) \bigr\Vert +\alpha_{n}\mu \Vert v \Vert . \end{aligned}$$

From Lemma 2.7, (48), and (49), we can conclude that

50 $$\begin{aligned} \bigl\Vert x_{n+1}-x^{*} \bigr\Vert =& (1-\alpha_{n}\tau) \bigl\Vert u_{n}-x^{*} \bigr\Vert +\alpha_{n}\mu \Vert v \Vert \\ \leq& (1-\alpha_{n}\tau) \bigl\Vert x_{n}-x^{*} \bigr\Vert +\alpha_{n}\tau \biggl(\frac{\mu \Vert v \Vert }{\tau} \biggr). \end{aligned}$$

This implies that

$$\bigl\Vert x_{n+1}-x^{*} \bigr\Vert \leq\max \biggl\{ \bigl\Vert x_{n}-x^{*} \bigr\Vert ,\frac{\mu \Vert v \Vert }{\tau } \biggr\} . $$

By induction, we obtain

$$\bigl\Vert x_{n}-x^{*} \bigr\Vert \leq\max \biggl\{ \bigl\Vert x_{0}-x^{*} \bigr\Vert ,\frac{\mu \Vert v \Vert }{\tau } \biggr\} . $$

Thus the sequence $\{x_{n}\}$ is bounded. Hence we can conclude from (48) and Lemma 4.3 that $\{y_{n}\}$ and $\{u_{n}\}$ are bounded, respectively. From condition (A4), we have $\{w_{n}\}$ is also bounded.

*Step 3*: We show that if there is a subsequence $\{ x_{n_{k}}\}$ of $\{x_{n}\}$ converging weakly to *x̄* and $\lim_{k\rightarrow\infty}(\sigma_{n_{k}}\|t_{n_{k}}\|)=0$, then we have $\bar{x}\in\varOmega$.

Firstly, we will show that $\{t_{n_{k}}\}$ is bounded. Since $\{ z_{n}\}$ is bounded, there is a subsequence $\{z_{n_{k}}\}$ of $\{z_{n}\}$ converging weakly to *z̄* By using Lemma 4.2, for any $\varepsilon>0$, there exist $\eta >0$ and $k_{0}$ such that

$$\partial_{2}g(z_{n_{k}},x_{n_{k}})\subset \partial_{2}g(\bar{z}, \bar {x})+\frac{\varepsilon}{\eta}B $$

for all $k\geq k_{0}$. Since $\{t_{n_{k}}\}\in\partial_{2}g(z_{n_{k}},x_{n_{k}})$, we have $\{t_{n_{k}}\}$ is bounded. Next, we show that $\| x_{n_{k}}-y_{n_{k}}\|\rightarrow0$. Without loss of generality, we can assume that $x_{n_{k}}\neq y_{n_{k}}$ for all $k \in\mathbb{N}$. By Lemma 4.1, we obtain $g(z_{n_{k}}, x_{n_{k}}) > 0$ and $t_{n_{k}}\neq0$. Since $\lim_{k\rightarrow\infty}(\sigma_{n_{k}}\| t_{n_{k}}\|)=0$ and $\{t_{n_{k}}\}$ is bounded, we have

51 $$ \lim_{k\rightarrow\infty}g(z_{n_{k}}, x_{n_{k}}) =\lim_{k\rightarrow\infty}\bigl(\sigma_{n_{k}} \Vert t_{n_{k}} \Vert \bigr) \Vert t_{n_{k}} \Vert =0. $$

It follows from the convexity of $g(z_{n_{k}},\cdot)$ that

$$ \gamma_{n_{k}}g(z_{n_{k}},y_{n_{k}})+(1- \gamma_{n_{k}})g(z_{n_{k}},x_{n_{k}}) \geq g(z_{n_{k}},z_{n_{k}})=0. $$

This implies that

52 $$ \gamma_{n_{k}} \bigl[g(z_{n_{k}},x_{n_{k}})-g(z_{n_{k}},y_{n_{k}}) \bigr] \leq g(z_{n_{k}},x_{n_{k}}). $$

By the Armijo line search, we get $\frac{\rho}{2\lambda_{n}}\|x_{n}-y_{n}\|^{2}\leq g(z_{n_{k}},x_{n_{k}})-g(z_{n_{k}},y_{n_{k}})$, and (52) implies that

53 $$ \frac{\rho\gamma_{n_{k}}}{2\lambda_{n_{k}}} \Vert x_{n}-y_{n} \Vert ^{2} \leq\gamma _{n_{k}} \bigl[g(z_{n_{k}},x_{n_{k}})-g(z_{n_{k}},y_{n_{k}}) \bigr] \leq g(z_{n_{k}},x_{n_{k}}). $$

Combining (51) with (53), we obtain

54 $$ \lim_{k\rightarrow\infty}\gamma_{n_{k}} \Vert x_{n}-y_{n} \Vert ^{2}=0. $$

Then we consider two cases.

Case 1. $\limsup_{k\rightarrow\infty}\gamma_{n_{k}}>0$. There exist $\bar{\gamma}>0 $ and a subsequence of $\{\gamma_{n_{k}}\}$ denoted again by $\{\gamma_{n_{k}}\}$ such that $\gamma_{n_{k}} > \bar{\gamma}$ for all *k*. So we get from (54) that

55 $$ \lim_{k\rightarrow\infty} \Vert x_{n_{k}}-y_{n_{k}} \Vert =0. $$

Since $x_{n_{k}}\rightharpoonup\bar{x}$ and (55), we have $y_{n_{k}}\rightharpoonup\bar{x}$. On the other hand, by the definition of $y_{n_{k}}$, we have

56 $$ \lambda_{n_{k}}\bigl\{ g(x_{n_{k}},y)-g(x_{n_{k}},y_{n_{k}}) \bigr\} \geq\langle x_{n_{k}}-y_{n_{k}},y- y_{n_{k}}\rangle \quad \text{for all } y\in C. $$

Therefore

$$\lambda_{n_{k}}\bigl\{ g(x_{n_{k}},y)-g(x_{n_{k}},y_{n_{k}}) \bigr\} \geq- \Vert x_{n_{k}}-y_{n_{k}} \Vert \Vert y- y_{n_{k}} \Vert \quad \text{for all } y\in C. $$

Letting $k\rightarrow\infty$ in the above inequality, using (55) and the jointly weak continuity of *g*, we have $g(\bar{x},y)-g(\bar{x},\bar{x})\geq0$ for all $y\in C$. So, $g(\bar{x},y)\geq0$ for all $y\in C$. Hence $\bar{x}\in\varOmega$.

Case 2. $\limsup_{k\rightarrow\infty}\gamma_{n_{k}}=0$. From the boundedness of $\{y_{n}\}$, there exists $\{y_{n_{k}}\}\subseteq\{y_{n}\}$ such that $y_{n_{k}}\rightharpoonup\bar{y}$. Let $\{m_{k}\}$ be the sequence of the smallest non-negative integers such that $z_{n_{k}}=(1-\gamma^{m_{k}})x_{n_{k}}+\gamma^{m_{k}}y_{n_{k}}$ and

$$g(z_{n_{k}}, x_{n_{k}})-g(z_{n_{k}},y_{n_{k}}) \geq \frac{\rho}{2\lambda_{n_{k}}} \Vert x_{n_{k}}-y_{n_{k}} \Vert ^{2}. $$

Since $\gamma^{m_{k}}\rightarrow0$, we have $m_{k}>0$. It follows from the Armijo line search that, for $m_{k}-1$, we have $\bar{z}_{n_{k}}=(1-\gamma^{m_{k}-1})x_{n_{k}}+\gamma^{m_{k}-1}y_{n_{k}}$ and

57 $$ g(\bar{z}_{n_{k}}, x_{n_{k}})-g( \bar{z}_{n_{k}},y_{n_{k}}) < \frac{\rho}{2\lambda_{n_{k}}} \Vert x_{n_{k}}-y_{n_{k}} \Vert ^{2}. $$

On the other hand, by the definition of $y_{n}$, we have

58 $$ \lambda_{n}\bigl\{ g(x_{n},y)-g(x_{n},y_{n}) \bigr\} \geq\langle x_{n}-y_{n},y- y_{n}\rangle \quad \text{for all } y\in C. $$

Letting $n=n_{k}$ and $y=x_{n_{k}}$ in (58), we get

59 $$ -\lambda_{n_{k}}g(x_{n_{k}},y_{n_{k}})\geq \Vert x_{n_{k}}-y_{n_{k}} \Vert ^{2}. $$

Combining (57) with (59), we obtain

60 $$ g(\bar{z}_{n_{k}}, x_{n_{k}})-g( \bar{z}_{n_{k}},y_{n_{k}})< -\frac{\rho }{2}g(x_{n_{k}},y_{n_{k}}). $$

Since $x_{n_{k}}\rightharpoonup\bar{x}, y_{n_{k}}\rightharpoonup\bar{y}$, and $\gamma_{n_{k}}\rightarrow0$, we have $\bar{z}_{n_{k}}\rightharpoonup \bar{x}$. From (60) and *g* is jointly weakly continuous on $H \times H$, we get $-g(\bar{x},\bar{y})<-\frac{\rho}{2}g(\bar{x},\bar{y})$. Since $\rho\in(0,2)$, we have $g(\bar{x},\bar{y})\geq0$. Taking $k\rightarrow\infty$ in (59), we obtain $\lim_{k\rightarrow\infty}\|x_{n_{k}}-y_{n_{k}}\|=0$. By Case 1, it is immediate that $\bar{x}\in\varOmega$.

*Step 4*: Show that the sequence $\{x_{n}\}$ converges strongly to $x^{*}$. By using the definition of $x_{n+1}$ and (44), we obtain

61 $$\begin{aligned} \bigl\Vert x_{n+1}-x^{*} \bigr\Vert ^{2} =& \bigl\Vert P_{C} \bigl(u_{n}-\alpha_{n}\mu w_{n}-P_{C} \bigl(x^{*}\bigr)\bigr) \bigr\Vert ^{2} \\ \leq& \bigl\Vert u_{n}-\alpha_{n}\mu w_{n}-x^{*} \bigr\Vert ^{2} \\ =& \bigl\Vert u_{n}-x^{*} \bigr\Vert ^{2}- 2\alpha_{n}\mu\bigl\langle w_{n},u_{n}-x^{*} \bigr\rangle + \bigl(\alpha_{n}\mu \Vert w_{n} \Vert \bigr)^{2} \\ \leq& \bigl\Vert x_{n}-x^{*} \bigr\Vert ^{2}-\xi_{n}(2-\xi_{n}) \bigl(\sigma_{n} \Vert t_{n} \Vert \bigr)^{2} \\ &{}- 2\alpha_{n}\mu\bigl\langle w_{n},u_{n}-x^{*} \bigr\rangle + \bigl(\alpha_{n}\mu \Vert w_{n} \Vert \bigr)^{2} \\ \leq& \bigl\Vert x_{n}-x^{*} \bigr\Vert ^{2}- \bigl(\sigma_{n} \Vert t_{n} \Vert \bigr)^{2} - 2\alpha_{n}\mu\bigl\langle w_{n},u_{n}-x^{*} \bigr\rangle \\ &{} + \bigl(\alpha_{n}\mu \Vert w_{n} \Vert \bigr)^{2}. \end{aligned}$$

Setting $a_{n}=\|x_{n}-x^{*}\|^{2}$. It follows from the boundedness of $\{w_{n}\}$ and $\{u_{n}\}$ that

62 $$ \bigl\vert \bigl\langle w_{n},u_{n}-x^{*} \bigr\rangle \bigr\vert \leq M_{1}\quad \text{and}\quad \Vert w_{n} \Vert ^{2}\leq M_{2}. $$

Combining (61), (62) with the definition of $a_{n}$, we get

63 $$ a_{n+1}-a_{n}+\bigl(\sigma_{n} \Vert t_{n} \Vert \bigr)^{2}\leq2\alpha_{n}\mu M_{1} +\alpha_{n}^{2}\mu^{2} M_{2}. $$

Let us consider two cases.

Case 1: There exists $n_{0}$ such that $\{a_{n}\}$ is decreasing for $n\geq n_{0}$. Therefore the limit of $\{a_{n}\}$ exists, denoted by *a*. It follows that $\lim_{n\rightarrow\infty}(a_{n+1}-a_{n})=0$. From (63) and the definition of $\alpha_{n}$, we have

64 $$ \lim_{n\rightarrow\infty}\bigl(\sigma_{n} \Vert t_{n} \Vert \bigr)=0. $$

By the definitions of $x_{n+1}$ and $u_{n}$, we obtain

65 $$\begin{aligned} \Vert u_{n}-x_{n} \Vert ^{2} =& \bigl\Vert P_{C}(x_{n}- \sigma_{n}t_{n})-P_{C}(x_{n}) \bigr\Vert \\ \leq& \Vert x_{n}-\sigma_{n}t_{n}-x_{n} \Vert \\ =& \sigma_{n} \Vert t_{n} \Vert . \end{aligned}$$

It follows that $\lim_{n\rightarrow\infty}\|u_{n}-x_{n}\|^{2}=0$, which implies that

66 $$ \lim_{n\rightarrow\infty} \bigl\Vert u_{n}-x^{*} \bigr\Vert ^{2}=a. $$

Since $\{u_{n}\}\subseteq C$ is bounded, there exists a subsequence $\{ u_{n_{k}}\}$ of $\{u_{n}\}$ that converges weakly to some $\bar{u}\in C$ and satisfies the equality

$$\liminf_{n\rightarrow\infty}\bigl\langle u_{n}-x^{*},v \bigr\rangle =\lim_{k\rightarrow\infty}\bigl\langle u_{n_{k}}-x^{*},v \bigr\rangle . $$

Since $u_{n_{k}}\rightharpoonup\bar{u}\in C$ and (43), we have

67 $$ \liminf_{n\rightarrow\infty}\bigl\langle u_{n}-x^{*},v \bigr\rangle =\lim_{k\rightarrow\infty}\bigl\langle u_{n_{k}}-x^{*},v \bigr\rangle =\bigl\langle \bar{u}-x^{*},v\bigr\rangle \geq0. $$

Since $w_{n}\in\partial_{2}f(u_{n},u_{n}), v\in\partial_{2}f(x^{*},x^{*})$ and *f* is *β*-strongly monotone on *C*, we have

$$\begin{aligned} \bigl\langle w_{n}, x^{*}-u_{n}\bigr\rangle \leq f\bigl(u_{n},x^{*}\bigr) \leq& -\beta \bigl\Vert u_{n}-x^{*} \bigr\Vert ^{2}-f \bigl(x^{*},u_{n}\bigr) \\ =& -\beta \bigl\Vert u_{n}-x^{*} \bigr\Vert ^{2}-\bigl\langle u_{n}-x^{*},v\bigr\rangle . \end{aligned}$$

This implies that $\langle w_{n}, u_{n}-x^{*}\rangle\geq\beta\|u_{n}-x^{*}\|^{2}+\langle u_{n}-x^{*},v\rangle$. Combining (66), (67) with the above inequality, we get

68 $$ \liminf_{n\rightarrow\infty}\bigl\langle w_{n},u_{n}-x^{*} \bigr\rangle \geq\beta a. $$

Assume that $a>0$. Choose $\varepsilon= \frac{1}{2}\beta a$. There exists $n_{0}$ such that

$$\bigl\langle u_{n}-x^{*}, w_{n}\bigr\rangle \geq \beta a-\varepsilon=\frac {1}{2}\beta a\quad \text{for all } n\geq n_{0}. $$

It follows from (61) and the above inequality that $a_{n+1}-a_{n}\leq-\alpha_{n}\mu\beta a^{2}+\alpha_{n}^{2}\mu M_{2}$. Summing this inequality from $n_{0}$ to *n*, we obtain

69 $$ a_{n+1}-a_{n_{0}}\leq-\mu\beta a^{2}\sum _{k=n_{0}}^{n}\alpha_{k} +\mu M_{2}\sum_{k=n_{0}}^{n} \alpha_{n}^{2}. $$

Since $\sum_{k=n_{0}}^{\infty}\alpha_{k}=\infty$ and $\sum_{k=n_{0}}^{\infty}\alpha_{n}^{2}<\infty$, we can conclude from (69) that $\liminf_{n\rightarrow\infty}a_{n}=-\infty$, which is a contradiction. So, $a=0$, and we can conclude that $\lim_{n\rightarrow\infty}\|x_{n}-x^{*}\|^{2}=0$.

Case 2: There exists a subsequence $\{a_{n_{j}}\}$ of $\{a_{n}\} $ such that $a_{n_{j}}\leq a_{n_{j}+1}$ for all $j\in\mathbb{N}$. Let $\{\tau(n)\}$ be a nondecreasing sequence defined in Lemma 2.6. Thus

70 $$ a_{\tau(n)}\leq a_{\tau(n)+1}\quad \text{and}\quad a_{n}\leq a_{\tau(n)+1}. $$

From (63) and the definition of $\alpha_{n}$, we have

71 $$ \lim_{n\rightarrow\infty}\sigma_{\tau(n)} \Vert t_{\tau(n)} \Vert =0. $$

By the definition of $u_{\tau(n)}$, we obtain

72 $$\begin{aligned} \Vert u_{\tau(n)}-x_{\tau(n)} \Vert ^{2} =& \bigl\Vert P_{C}(x_{\tau(n)}- \sigma_{\tau (n)}t_{\tau(n)})-P_{C}(x_{\tau(n)}) \bigr\Vert \\ \leq& \Vert x_{\tau(n)}-\sigma_{\tau(n)}t_{\tau(n)}-x_{\tau(n)} \Vert \\ =& \sigma_{\tau(n)} \Vert t_{\tau(n)} \Vert . \end{aligned}$$

This implies that $\lim_{n\rightarrow\infty}\|u_{\tau(n)}-x_{\tau(n)}\| ^{2}=0$. Since $\{x_{\tau(n)}\}$ is bounded, there exists a subsequence $\{x_{{\tau(n)}_{k}}\}\subseteq\{x_{\tau(n)}\}$ such that $x_{{\tau(n)}_{k}}\rightharpoonup\bar{x}\in C$ and thus $u_{{\tau(n)}_{k}}\rightharpoonup\bar{x}\in C$. From (71) and Step 3 of this proof, we get $\bar{x}\in\varOmega$. Next, we will show that $u_{\tau(n)_{k}}\rightarrow x^{*}$. It follows from (61) that

$$\begin{aligned} 2\alpha_{\tau(n)_{k}}\mu\bigl\langle w_{\tau(n)_{k}},u_{\tau (n)_{k}}-x^{*} \bigr\rangle \leq& a_{\tau(n)_{k}}-a_{{\tau(n)_{k}}+1}-\bigl(\sigma _{{\tau(n)}_{k}} \Vert t_{{\tau(n)}_{k}} \Vert \bigr)^{2} \\ & {}+\bigl(\alpha_{{\tau(n)}_{k}}\mu \Vert w_{{\tau(n)}_{k}} \Vert \bigr)^{2}\leq(\alpha_{{\tau (n)}_{k}}\mu)^{2}M_{2}. \end{aligned}$$

This implies that

73 $$ \bigl\langle w_{\tau(n)_{k}},u_{\tau(n)_{k}}-x^{*} \bigr\rangle \leq\frac{\alpha _{\tau(n)_{k}}\mu M_{2}}{2}. $$

Since *f* is *β*-strongly monotone on *C* and $w_{\tau(n)}\in \partial_{2}f(u_{\tau(n)_{k}},u_{\tau(n)_{k}})$, we have

$$\bigl\langle x^{*}-u_{\tau(n)_{k}}, w_{\tau(n)_{k}}\bigr\rangle \leq f\bigl(u_{\tau (n)_{k}},x^{*}\bigr)\leq-\beta \bigl\Vert u_{\tau(n)_{k}}-x^{*} \bigr\Vert ^{2}-f \bigl(x^{*},u_{\tau (n)_{k}}\bigr). $$

It follows from (73) and the above inequality that

74 $$\begin{aligned} \bigl\Vert u_{\tau(n)_{k}}-x^{*} \bigr\Vert ^{2} \leq& \frac{1}{\beta} \bigl[ \bigl\langle w_{\tau(n)_{k}},u_{\tau(n)_{k}}-x^{*}\bigr\rangle -f \bigl(x^{*},u_{\tau (n)_{k}}\bigr) \bigr] \\ \leq& \frac{1}{\beta} \biggl[ \frac{\alpha_{\tau(n)_{k}}\mu M_{2}}{2}-f\bigl(x^{*},u_{\tau(n)_{k}} \bigr) \biggr]. \end{aligned}$$

Taking $k\rightarrow\infty$, by using $u_{\tau(n)_{k}}\rightharpoonup \bar{x}$ and $\alpha_{\tau(n)_{k}}\rightarrow0$, we get

$$\limsup_{k\rightarrow\infty} \bigl\Vert u_{\tau(n)_{k}}-x^{*} \bigr\Vert ^{2}\leq -f\bigl(x^{*},\bar{x}\bigr)\leq0. $$

Therefore $\lim_{k\rightarrow\infty}\|u_{\tau(n)_{k}}-x^{*}\|^{2}=0$. Then, it is easy to see that $\lim_{n\rightarrow\infty}\|u_{\tau(n)}-x^{*}\|^{2}=0$. By the definition of $x_{n+1}$, we have

75 $$\begin{aligned} \bigl\Vert x_{\tau(n)+1}-x^{*} \bigr\Vert \leq& \bigl\Vert u_{\tau(n)}-\alpha_{\tau(n)}\mu w_{\tau(n)}-x^{*} \bigr\Vert \\ \leq& \bigl\Vert u_{\tau(n)}-x^{*} \bigr\Vert + \alpha_{\tau(n)}\mu \Vert w_{\tau(n)} \Vert . \end{aligned}$$

Since $\{w_{\tau(n)}\}$ is bounded, and from the definition of $\alpha _{\tau(n)}$, we have $\lim_{n\rightarrow\infty}\|x_{\tau(n)+1}-x^{*}\|^{2}=0$. This means that $\lim_{n\rightarrow\infty}a_{\tau(n)+1}=0$. It follows from (50) that $\lim_{n\rightarrow\infty}a_{n}=0$. Hence $\lim_{n\rightarrow\infty}\|x_{n}-x^{*}\|^{2}=0$. This completes the proof. □

## Numerical examples

Let $H=\mathbb{R}^{n}$ and $C= \{x\in\mathbb{R}^{n}:-5 \leq x_{i}\leq 5,\forall i\in\{1,2,\ldots,n\}\}$. Let the bifunction $g: \mathbb {R}^{n}\times\mathbb{R}^{n}\to\mathbb{R}$ be defined by

$$g(x,y)= \langle Px+Qy, y-x\rangle\quad \text{for all } x,y\in \mathbb{R}^{n}, $$

where *P* and *Q* are randomly symmetric positive semidefinite matrices such that $P-Q$ is positive semidefinite. Then *g* is pseudomonotone on $\mathbb{R}^{n}$. Indeed, let $g(x,y)\geq0$ for every $x,y\in\mathbb{R}^{n}$, we have

$$\begin{aligned} g(y,x)\leq g(x,y)+g(y,x) =& \langle P x+ Q y, y-x\rangle+\langle P y+ Q x, x-y\rangle \\ =& -\bigl\langle (P-Q) (x-y), x-y\bigr\rangle \leq0. \end{aligned}$$

Next, we obtain that *g* is Lipschitz-type continuous with $L_{1}=L_{2}=\frac{1}{2}\|P-Q\|$. Indeed, for each $x,y,z \in\mathbb{R}^{n}$,

$$\begin{aligned} g(x,y)+g(y,z)-g(x,z) =&\langle P x + Q y, y-x\rangle+\langle P y+ Q z, z-y\rangle-\langle P x + Q z , z-x\rangle \\ =& \bigl\langle (P-Q) (x-y), y-z\bigr\rangle \\ \geq& -2\frac{ \Vert P-Q \Vert }{2} \Vert x-y \Vert \Vert y-z \Vert \\ \geq& -\frac{ \Vert P-Q \Vert }{2} \Vert x-y \Vert ^{2}-\frac{ \Vert P-Q \Vert }{2} \Vert y-z \Vert ^{2}, \end{aligned}$$

where $\|P-Q\|$ is the spectral norm of the matrix $\|P-Q\|$, that is, the square root of the largest eigenvalue of the positive semidefinite matrix $(P-Q)^{T}(P-Q)$. It is easy to check that $\varOmega\neq\emptyset$. Furthermore, we define the bifunction $f:\mathbb{R}^{n}\times\mathbb{R}^{n}\to\mathbb{R}$ as

$$f(x,y)= \langle Ax +By, y-x\rangle\quad \text{for all } x,y\in \mathbb{R}^{n}, $$

with *A* and *B* being positive definite matrices defined by

$$B = N^{T}N + n I_{n}\quad \text{and}\quad A = B + M^{T}M+ n I_{n}, $$

where $M,N$ are randomly $n\times n$ matrices and $I_{n}$ is the identity matrix. Then we have *f* is *n*-strongly monotone on $\mathbb{R}^{n}$. Indeed, let $x,y\in\mathbb{R}^{n}$, we get

$$\begin{aligned} f(x,y)+f(y,x) =& \langle A x + B y, y-x\rangle+ \langle A y + B x, x-y\rangle \\ =& -\bigl\langle (A-B) (x-y), x-y\bigr\rangle \\ =& -\bigl\langle M^{T}M+ n I_{n}(x-y), x-y\bigr\rangle \\ =& -\bigl\langle M^{T}M (x-y), x-y\bigr\rangle -\bigl\langle n I_{n}(x-y), x-y\bigr\rangle \\ =& - \bigl\Vert M(x-y) \bigr\Vert ^{2}-n \Vert x-y \Vert ^{2} \\ \leq& -n \Vert x-y \Vert ^{2}. \end{aligned}$$

Moreover, $\partial_{2}f(x,x)=\{(A+B) x\}$ and $\|(A+B)x - (A+B)y\|\leq\|A+B\|\|x-y\|$ for all $x,y\in\mathbb{R}^{n}$. Thus the mapping $x \rightarrow\partial_{2}g(x,x)$ is bounded and $\| A+B\|$-Lipschitz continuous on every bounded subset of *H*. In this example, we consider the quadratic optimization

76 $$ \min_{x\in C} \biggl\{ \frac{1}{2}x^{T}Hx+f^{T}x \biggr\} , $$

where *H* is a matrix, *f* and *x* are vectors. From the subproblem of solving $y_{k}$ and $z_{k}$ in Algorithm 1, we can consider problem (76).

We have tested for this example where $n=5, 10, 50,100$, and 500. Starting point $x_{0}$ is a randomly initial point. Take the parameters

$$\alpha_{k}=\frac{1}{k+4},\qquad \eta_{k}= \frac{k+1}{3(k+4)},\qquad \lambda_{k}=\frac{1}{2 \Vert P-Q \Vert },\qquad \mu= \frac{2}{ \Vert A+B \Vert ^{2}}. $$

We have implemented Algorithm 1 for this problem in Matlab R2015 running on a Desktop with Intel(R) Core(TM) i5-7200u CPU 2.50 GHz, and 4 GB RAM, and we used the stopping criteria $\|x_{k+1} - x_{k}\|<\varepsilon $ with $\varepsilon= 0.001$ is a tolerance to cease the algorithm. Denote that

- N.P: the number of the tested problems.
- Average iteration: the average number of iterations.
- Average times: the average CPU-computation times (in s).

The computation results are reported in the following tables.

From the numerical result Table 1, we see that the sequence generated by our algorithms is convergent and effective for solving the solution of bilevel equilibrium problems.

**Table 1:** The results computed on Algorithm 1

*n*	N.P.	Average iteration	Average times
5	10	72	0.7219
10	10	255	2.3859
50	10	1224	12.6235
100	10	1541	37.8766
500	10	1910	465.510

## Conclusions

We have proposed two iterative algorithms for finding the solution of a bilevel equilibrium problem in a real Hilbert space. The sequence generated by our algorithms converges strongly to the solution. Furthermore, we reported the numerical result to support our algorithm.
